# Correlation between two- and three-dimensional crystallographic lattices for epitaxial analysis. I. Theory

**DOI:** 10.1107/S2053273322002182

**Published:** 2022-04-11

**Authors:** Josef Simbrunner, Jari Domke, Roman Forker, Roland Resel, Torsten Fritz

**Affiliations:** a Division of Neuroradiology, Vascular and Interventional Radiology, Medical University Graz, Auenbruggerplatz 9, Graz, 8036, Austria; b Institute of Solid State Physics, Friedrich Schiller University Jena, Helmholtzweg 5, Jena, 07743, Germany; c Institute of Solid State Physics, Graz University of Technology, Petersgasse 16, Graz, 8010, Austria

**Keywords:** crystallographic lattices, surface unit cell, GIXD, mathematical crystallography, thin films

## Abstract

The epitaxial growth of molecular crystals at single-crystalline surfaces is often strongly related to the first monolayer at the substrate surface. The present work presents a theoretical approach to compare three-dimensional lattices of epitaxially grown crystals with two-dimensional lattices of the molecules formed within the first monolayer. Real-space and reciprocal-space representations are considered. Depending on the crystallographic orientation relative to the substrate surface, proper linear combinations of the lattice vectors of the three-dimensional unit cell result in a rhomboid in the *xy* plane, representing a two-dimensional projection. Mathematical expressions are derived which provide a relationship between the six lattice parameters of the three-dimensional case and the three parameters obtained for the two-dimensional surface unit cell. It is found that rotational symmetries of the monolayers are reflected by the epitaxial order. Positive and negative orientations of the crystallographic contact planes are correlated with the mirror symmetry of the surface unit cells, and the corresponding mathematical expressions are derived. The method is exemplarily applied to data obtained in previous grazing-incidence X-ray diffraction (GIXD) measurements with sample rotation on thin films of the conjugated molecules 3,4;9,10-perylenetetra­carb­oxy­lic dianhydride (PTCDA), 6,13-pentacene­quinone (P2O), 1,2;8,9-dibenzopentacene (*trans*-DBPen) and di­cyano­vinyl-quaterthio­phene (DCV4T-Et2) grown by physical vapor deposition on Ag(111) and Cu(111) single crystals. This work introduces the possibility to study three-dimensional crystal growth nucleated by an ordered monolayer by combining two different experimental techniques, GIXD and low-energy electron diffraction, which has been implemented in the second part of this work.

## Abstract 2

A general formalism to determine the surface unit cell of a three-dimensional crystallographic lattice is presented.

## 1. Introduction

Crystal structure identification of thin organic films attracts considerable interest in organic electronics and pharmaceutical science (Jones *et al.*, 2016 ▸). The presence of a single-crystalline surface during the crystallization process can induce new types of molecular packing because the substrate acts as template for the crystallization process. In the following, some selected examples are given. In pentacene/Cu(110) molecular reorientation from planar adsorption geometry towards an upright orientation was observed (Koini *et al.*, 2008 ▸; Lukas *et al.*, 2004 ▸, Söhnchen *et al.*, 2004 ▸). In *para*-sexi­phenyl/KCl(001) epitaxial growth was found with four contact planes (Haber *et al.*, 2005 ▸). In platelets of the same molecule on a series of alkali halide surfaces a variety of preferential orientations were observed and analyzed (Smilgies & Kintzel, 2009 ▸). In general, if molecular crystals are epitaxially grown on single-crystalline substrates, multiple preferred orientations of the adsorbate, several symmetry-related in-plane alignments, and the occurrence of unknown polymorphs can be observed (Mitchell *et al.*, 2001 ▸; Resel *et al.*, 2009 ▸; Simbrunner *et al.*, 2011 ▸; Schwabegger *et al.*, 2013 ▸).

Crystal structure identification from thin films is often performed by grazing-incidence X-ray diffraction (GIXD) experiments. The analysis of the diffraction pattern relies on indexing the obtained reflections to determine the unit cells formed by the underlying molecules. In a previous work, we described an algorithm which proved effective for the thin film analysis, where unit cells in various orientations and/or with different lattice parameters may occur (Simbrunner *et al.*, 2020 ▸, 2021*a* ▸). The following general crystallographic features of epitaxially grown films could be observed: (i) the crystallites grow with defined crystallographic planes parallel to the substrate surface (*i.e.* contact planes), which can be observed by specular X-ray diffraction. The specular diffraction peak comprises the information on the Miller indices of the contact plane. In all our test cases, we found positive and negative orientations of the contact planes, *i.e*. the planes with the Miller indices (*uvw*) and ( 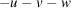 ). In the case of di­cyano­vinyl-quaterthio­phene (DCV4T-Et2) on Ag(111) we observed three polymorphs with both different crystallographic unit cells as well as contact planes (Simbrunner *et al.*, 2021*a* ▸). (ii) The crystallites show additionally distinct rotational alignments in the *xy* plane. For each contact plane two groups of 60° symmetry were observed, one for the positive (*uvw*) and one for the negative ( 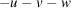 ) orientation. The respective two main axes of the organic crystals are aligned symmetrically, mostly anticlockwise and clockwise, with respect to the main axes of the substrates. Hence, GIXD is a very powerful experimental method to study the three-dimensional crystal structure of thin, organic, epitaxially grown films.

However, for studying epitaxial growth, the molecular contact layer *(i.e*. first monolayer) and its relation to the substrate has gained special interest. The details of the adsorption, bonding and ordering of the first layer on the substrate surface can strongly determine the structure and morphology of the organic film growing on top. To elucidate the structure of the monolayer, two-dimensional surface-sensitive methods, such as distortion-corrected low-energy electron diffraction (LEED) (Sojka *et al.*, 2013*a* ▸,*b* ▸), are especially suited. The relationship between substrate and monolayer, which can be mathematically expressed in the epitaxy matrix, is of special importance (Kasemann *et al.*, 2009 ▸; Forker *et al.*, 2017 ▸).

Therefore, for studying epitaxial growth mechanisms, it is desirable to correlate the results of LEED and GIXD measurements to compare the crystallographic structures of the monolayer and the multilayer. In this work, we derive analytical mathematical expressions to correlate the parameters of an arbitrary three-dimensional lattice with those of its surface unit cell. To illustrate the applicability, the developed theoretical framework is applied in Section 3 to the conjugated molecules 6,13-pentacene­quinone (P2O), 3,4;9,10-perylenetetra­carb­oxy­lic dianhydride (PTCDA), 1,2;8,9-dibenzopentacene (*trans*-DBPen) and DCV4T-Et2, grown by physical vapor deposition on single-crystalline surfaces like Ag(111) and Cu(111), which we previously studied using rotated GIXD experiments (Simbrunner *et al.*, 2020 ▸, 2021*a* ▸). The two-dimensional lattice parameters are theoretically interpreted on the basis of the known three-dimensional unit cell and orientation parameters.

In the second part of our work (Simbrunner *et al.*, 2022 ▸), the analysis will be based upon the indexing of the two-dimensional diffraction patterns incorporated in the GIXD data, *i.e*. the *x* and *y* components of the reciprocal-lattice vectors, of the examples listed above, and obtained in LEED experiments on the same molecules.

## 2. Method

### 2.1. From three- to two-dimensional crystallographic lattices

For the following mathematical treatment, a crystal-fixed Cartesian coordinate system (laboratory system) is assumed, where the *xy* plane runs parallel to the substrate surface; *a*, *b*, *c*, α, β and γ are the parameters of the (direct) unit cell. It is convenient to arrange its lattice vectors **a**, **b** and **c** in the matrix **A** as follows:

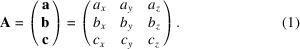

The absolute value of the determinant of **A** corresponds to the volume of the parallelepiped spanned by the lattice vectors. Then, the reciprocal-lattice vectors a*, b* and c* are given by the relation

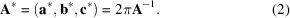

The reciprocal-lattice vector **g** with its Laue indices *h*, *k* and *l* can be represented by the equation

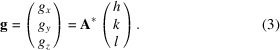

We assign the composite vectors 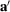 and 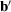 of the lattice vectors **a**, **b** and **c** such that they are confined to the *xy* plane, *i.e.*


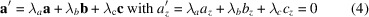

and

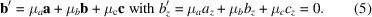

The vectors 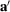 and 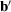 enclose the angle γ′, and their lengths are 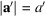 and 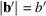 , respectively. Together with a composite vector 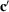 (which must contain a non-zero *z* component) 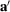 and 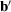 span a supercell. Its matrix 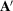 can be written as follows:

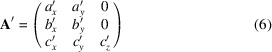

with

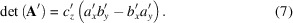

Then, the matrix 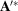 of the corresponding reciprocal-lattice vectors is given as follows:

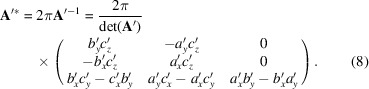




In the following, we will consider only the reciprocal vector 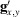 in the *xy* plane, which can be explicitly written as

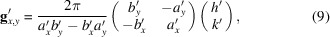

where 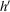 and 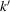 are the corresponding Laue indices. When the Laue condition, *i.e*. (*g_x_, g_y_*) = scattering vector (*q_x_, q_y_*), is fulfilled, diffraction in the *xy* plane can be observed.

For the following discourse, we will consider crystallographic unit cells with a contact plane characterized by the Miller indices *u*, *v* and *w*, which are integers (Simbrunner *et al.*, 2018 ▸). Then, the *z* components of the lattice vectors can be written as

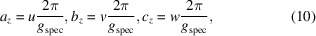

where *g*_spec_ corresponds to the height of the specular diffraction peak, which can be explicitly written as

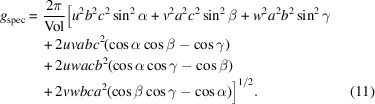

Vol is the unit-cell volume, which can be expressed as

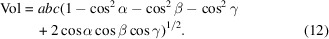

With the expressions in equation (10), the relations in equations (4) and (5) can be rewritten as

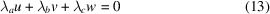

and

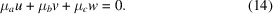

From these scalar vector products, the following cross product can be derived:

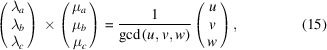

where gcd(*u*, *v*, *w*) is the greatest common divisor of the Miller indices. Note that for (*uvw*) → −(*uvw*) the coefficients are transformed as: (λ_*a*_, λ_*b*_, λ_*c*_) → (λ_*a*_, λ_*b*_, λ_*c*_) and (μ_*a*_, μ_*b*_, μ*_c_*) → −(μ_*a*_, μ_*b*_, μ_*c*_). The factors λ_*i*_ and μ_*i*_ can be regarded as components of the transformation matrix **N**, which linearly transforms the matrix **A** into **A**′, *i.e.* **A**′ = **NA** (Simbrunner *et al.*, 2018 ▸). Therefore, the following relations for the Laue indices *h*, *k* and *l* are valid:

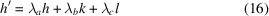

and

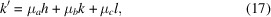

where *h*′ and *k*′ are the Laue indices of the supercell [*cf.* equation (9)]. Therefore, in general, λ_*i*_ and μ_*i*_ must be integers. For the lattice vectors 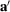 and 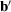 the following relations can be derived:

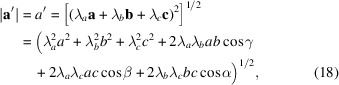




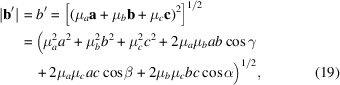




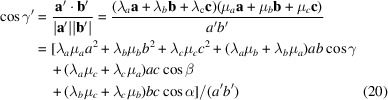

and

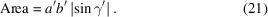




Furthermore [see Appendix *A* [App appa], equation (61)], the following expression for the area of the (reduced) rhomboid is valid:

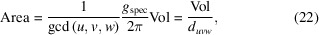

where *d_uvw_* is the interplanar distance. This relation is obvious, if, *e.g*. *u* = *v* = 0 and *w* = 1, where 

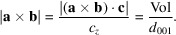

Equation (22) is, however, valid for any contact plane (*uvw*). For a further algebraic step, equation (11) could be used.

The vectors 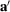 and 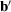 can be explicitly written as

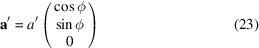

and

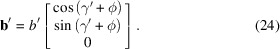




The derivation of the vector components of 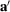 and 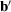 and the explicit expression for the angle ϕ, which is an elaborate function of the parameters of the three-dimensional unit cell and the coefficients λ_*i*_ and μ_*i*_, can be found in Appendix *A* [App appa]. Hence, equation (9) can be rewritten as follows:

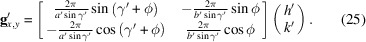

The reduced cell (rhomboid) is obtained by choosing the coefficients λ*_i_* and μ*_i_* so that the two shortest vectors, which are not collinear, result.

From equation (20) it can be deduced that for (*uvw*) → −(*uvw*) the following angular transformation results: γ′ → π − γ′. In equation (25), this is equivalent to γ′ → −γ′ and *h*′ → −*h*′, which corresponds to a mirror symmetry about an axis along the lattice vector 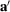 .

If *u*, *v* and *w* ≠ 0, using equations (4), (5) and (15), equations (18)–(20) can be transformed to the following relations:

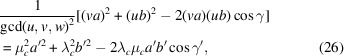




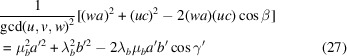

and

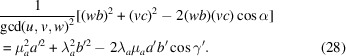

The expressions on the left side of the equations represent the squares of diagonals of the parallelepiped representing the three-dimensional unit cell or one of its supercells, depending upon the Miller indices of the contact plane. Further relations are derived in Appendix *B* [App appb].

### 2.2. Two-dimensional crystallographic lattice

For convenience, in the following paragraphs of Section 2 we will omit the prime for the parameters of the two-dimensional unit cell. The real-space lattice vectors **a** and **b** can be represented by the matrix **A**, which is explicitly written as

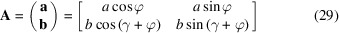

with the relations |**a**| = *a*, |**b**| = *b*, **a**  **b**/(*ab*) = 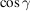 and det(**A**) = Area = 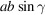 ; the angle φ represents a phase shift in the *xy* plane counter-clockwise. The reciprocal-lattice vectors are contained in the matrix A* as follows:

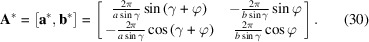

Equations (29) and (30) are connected via

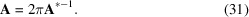

Then the two-dimensional reciprocal-lattice vector **g** with its Laue indices *h* and *k* is given by

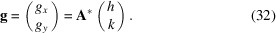

It can be easily recognized that equation (32) is equivalent to equation (25). Therefore, indexing LEED patterns technically corresponds to indexing GIXD patterns, when only the information in the *xy* plane (*i.e*. pairs of *q_x_* and *q_y_*) is used. When the Laue condition is fulfilled, *i.e*. the scattering vector in the *xy* plane **q**_*xy*_ = **g**, diffraction can be observed. Using equations (31) and (32), the following relation can be derived:

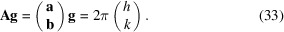

From equation (33) it can be deduced that, if two reciprocal vectors **g**_1_ and **g**_2_ are given, the following relation is valid:

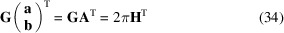

where

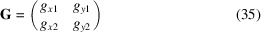

and ( 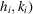 are the corresponding pairs of Laue indices with

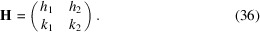




Equation (34) can be equivalently expressed as

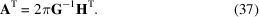

The unit-cell vectors must be solutions to all reciprocal vectors **g**_*i*_, which, according to equation (33), can be written as

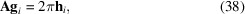

where **g**_*i*_ = (*g_xi_*, *g_yi_*)^T^ and **h**_*i*_ = (*h_i_*, *k_i_*)^T^. For a phase shift of 180° of either the lattice vectors **a** and **b** [*i.e.* φ → φ + π in equation (30)] or the reciprocal vector **g**_*i*_, **h**_*i*_ will become −**h**_*i*_. From equation (38) it can be deduced that 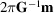 , the product of the inverse matrix of two reciprocal vectors with a vector **m**, consisting of a doublet of arbitrary integers (*m*_1_, *m*_2_), leads to a vector of the reduced cell, if **m** matches (*h*_1_, *h*_2_)^T^ or (*k*_1_, *k*_2_)^T^. If a transformation matrix **N** exists so that **m** equals **N**(*h*_1_, *h*_2_)^T^ or **N**(*k*_1_, *k*_2_)^T^, a vector of a superlattice is obtained. The reduced rhomboid is obtained by choosing the two shortest vectors which are not collinear and whose scalar products with all reciprocal vectors yield integers (Simbrunner *et al.*, 2021*b* ▸). This reduced rhomboid is equivalent to the Buerger cell in the three-dimensional case (Buerger, 1957 ▸). Analogously, the Niggli criteria (Niggli, 1928 ▸) can be expressed in the two-dimensional case as

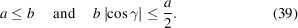




## 3. Application to specific molecule–substrate combinations

### 3.1. General remarks

To practically demonstrate the theoretical framework, we apply the developed mathematical approach to the epitaxially grown crystallites, *i.e*. P2O/Ag(111), PTCDA/Ag(111), DCV4T-Et2/Ag(111) and *trans*-DBPen/Cu(111) (Simbrunner *et al.*, 2020 ▸, 2021*a* ▸). Keeping with the notation in the theoretical part of our work in Section 2.1, again the components of the two-dimensional unit cells will be indicated by the prime.

In Table 1 ▸, the general equations (18)–(20) for calculating the cell parameters *a*′, *b*′ and γ′ from the corresponding parameters of the three-dimensional unit cell are applied to the four molecules. The coefficients λ_*i*_ and μ*_i_* are chosen such that equation (15) is fulfilled for the corresponding contact planes, indicated by the Miller indices *u*, *v* and *w*. As we have shown, the positive (*uvw*) and the negative ( 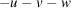 ) orientations of the contact plane correspond to the observed mirror symmetry of the two-dimensional unit cells (Kilian *et al.*, 2004 ▸; Simbrunner *et al.*, 2022 ▸). If the contact plane is perpendicular to one of the main axes of the Cartesian system, the two-dimensional unit cell is spanned by two vectors of the three-dimensional unit cell. Otherwise, the equations comprise parameters of all three dimensions. Elaborate relations arise, if none of the Miller indices of the contact plane is zero, as in the case of two polymorphs of DCV4T-Et2/Ag(111). In Table 2 ▸, the previously obtained three-dimensional parameters of the four examples are listed (Simbrunner *et al.*, 2020 ▸, 2021*a* ▸). In Table 3 ▸, these parameters are used to specifically calculate the corresponding parameters of the two-dimensional unit cells. In Table 4 ▸, the areas of the two-dimensional unit cells are calculated from the volumes of the three-dimensional unit cells and the respective lengths of the specular diffraction peaks obtained by X-ray diffraction, *g*_spec_ [*cf*. equation (22)]. Comparing the values of the areas with those in Table 3 ▸ [calculated with equation (21)] shows their accordance within the experimental error. In the second part of our work (Simbrunner *et al.*, 2022 ▸), we will determine the parameters of the surface unit cells experimentally by indexing our GIXD data again, using only the *x* and *y* components of the reciprocal-space vectors. We will see that such obtained results are in good accordance with the theoretically predicted data.

In the following paragraphs, for two molecules, *i.e*. PTCDA/Ag(111) and DCV4T-Et2/Ag(111), we will go into further depth.

### 3.2. PTCDA/Ag(111)

In the three-dimensional GIXD experiment, for the unit cell two groups of azimuthal alignments, each with a 60° symmetry, were found (Simbrunner *et al.*, 2021*a* ▸). These could be explained by the two contact planes (103) and (103). The orientation of the contact plane is usually indicated as (102) – for the reason of crystallographic convention, however, it is in the monoclinic system with the supplementary angle β > 90° (103) (Simbrunner *et al.*, 2021*a* ▸). As in the particular case of PTCDA the conditions *v* = 0 and α = γ = 90° are fulfilled, the lattice vectors **a**, **b**, **c** for the contact planes (103) and (103) are collinear; therefore, an unambiguous assignment of the rotation angles φ to either one of those contact planes is not possible (Simbrunner *et al.*, 2021*a* ▸).

In general, for a unit cell in (103) orientation, γ′ can be calculated as follows [*cf*. equation (20)]:

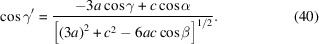

Then, for a monoclinic lattice (α = γ = 90°) γ′ = 90°. Accordingly, a rectangular surface unit cell can be observed in the multilayer (see Table 3 ▸). However, as in the LEED experiment the angle γ′ is about 89° (Kilian *et al.*, 2004 ▸; Simbrunner *et al.*, 2022 ▸), the two-dimensional unit cell in the monolayer is not rectangular. This demonstrates that the monolayer structure differs qualitatively from the monoclinic bulk lattice, although the quantitative difference is subtle.

### 3.3. DCV4T-Et2/Ag(111)

In the rotated GIXD experiment performed previously, we found three polymorphs with the contact planes ±(122), ±(211) and ±(020) (Simbrunner *et al.*, 2021*a* ▸; see Table 2 ▸). Theoretical considerations show that for the (122) orientation, equation (15) gives two solutions: in both cases λ_*b*_ = λ_*c*_ = −1, in (i) μ*_a_* = 2, μ*_b_* = 1 and in (ii) μ*_a_* = 2, μ*_b_* = 2, μ*_c_* = 1. Taking our data from the rotating GIXD experiment, the following parameters can be calculated: (i) *a*′ = 11.907, *b*′ = 16.849 Å, γ′ = 78.00° and (ii) *a*′ = 11.907, *b*′ = 18.500 Å, γ′ = 117.02°. For both solutions the area is 196.2 Å^2^. Solution (i), however, is the reduced Buerger cell.

For the (211) orientation, equation (15) gives only the solution λ_*b*_ = λ_*c*_ = μ_*c*_ = −1 and μ_*a*_ = μ_*b*_ = 1.

As for both contact planes none of the Miller indices is zero, no basis vector of the three-dimensional unit cell can be directly observed in the two-dimensional lattice; however, we can extract three diagonals of the parallelepiped, which are spanned by different vectors [*cf*. equations (26)–(28)]. In Table 5 ▸, we summarize the results of this analysis. This manifests that there is a clear relationship between the two lattices.

**Figure 1:**
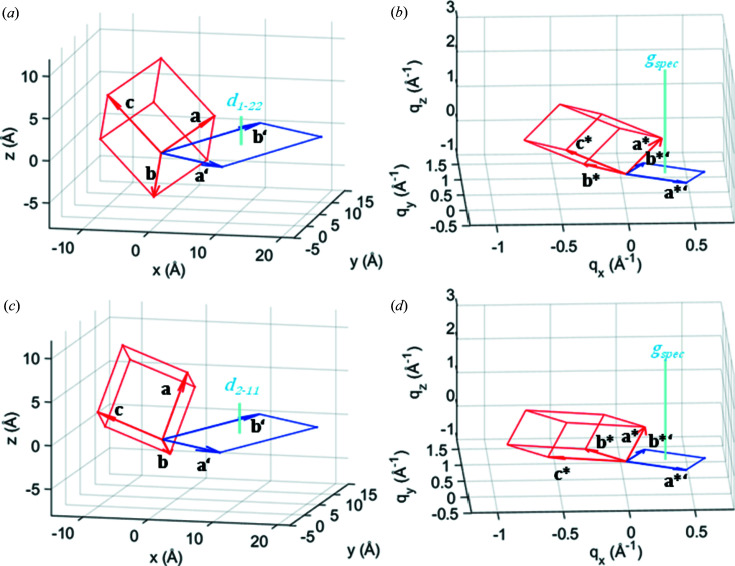
Schematic three-dimensional (red) and two-dimensional (blue) unit cells for the polymorphs of DCV4T-Et2/Ag(111) with (122) (*a*), (*b*) and (211) (*c*), (*d*) orientations. In (*a*), (*c*) the relations in the real, and in (*b*), (*d*) in the reciprocal space are depicted. Also shown (cyan) is the peak of the reciprocal scan *g*_spec_ in the reciprocal space and *d_uvw_* = 2π/ *g*_spec_ in the real space, respectively. Note that the volumes of the (red) parallelepipeds equal the products of the areas of the (blue) rhomboids with *d_uvw_* (*a*), (*c*) and *g*_spec_ (*b*), (*d*), respectively.

In Fig. 1 ▸, the schematic three- and two-dimensional unit cells of these two polymorphs in the real (*a*), (*c*) and in the reciprocal (*b*), (*d*) space are shown. Note that, whereas the parallelepipeds look different, the two-dimensional unit cells (rhomboids) are similar.

The parameters of the unit cell in the ±(020) orientation are shown in Tables 3 ▸ and 4 ▸. There is a certain relationship between 2*a* and *c* with the corresponding parameters *a*′ and *b*′ of the other two unit cells of DCV4T-Et2/Ag(111).

Furthermore, in the *xy* plane, these three polymorphs form two groups of related azimuthal alignments, each with a 60° symmetry and corresponding to the respective positive and negative contact planes (see Appendix *A* [App appa] and Table 6 ▸).

Hence, though the cell parameters and orientations of the three polymorphs are quite different in three dimensions (see Table 2 ▸), the respective parameters in the *xy* plane converge.

## 4. Summary and conclusion

For epitaxial analysis, it is desirable to determine the crystallographic lattices in the monolayer and in the multilayer. Analytical methods for the three-dimensional crystal structure, such as rotational GIXD, are able to provide spatial information. The monolayer, however, is only accessible in two dimensions, where distortion-corrected LEED is the method of choice.

A comprehensive mathematical framework has been developed to correlate the parameters of the two- and three-dimensional lattices. Knowing the orientation and parameters of the three-dimensional unit cell enables the interpretation of the two-dimensional data for direct comparison of the lattices. Depending upon the Miller indices of the contact plane, either basis vectors of the three-dimensional unit cell or composites of them build up the corresponding two-dimensional surface unit cell (rhomboid). The derived mathematical formulas have been applied on previously obtained GIXD data from various molecules on substrates such as Ag(111) and Cu(111). For lattices with orientations where all Miller indices are non-zero, as in the case of DCV4T-Et2/Ag(111), no vector of the surface unit cell is a basis vector of the three-dimensional lattice. There is, however, access to three diagonals of different planes of the three-dimensional unit cell or one of its supercells.

In previous GIXD experiments, for the three-dimensional unit cells we found positive and negative orientations of the contact planes, *i.e*. the planes with the Miller indices (*uvw*) and ( 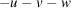 ). Furthermore, distinct alignments of the crystallites in the *xy* plane were found, *i.e*. for each contact plane two groups of 60° symmetry were observed, one for the positive (*uvw*) and one for the negative ( 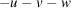 ) orientation. In this study we demonstrate that in two dimensions this feature corresponds to unit cells with mirror symmetry with respect to the *a* axis. Thus, rotational and mirror symmetries coexist.

In a previous study, in the multilayer of DCV4T-Et2/Ag(111), we found three polymorphs with various cell parameters, orientations and azimuthal alignments. The theoretical analysis of the corresponding two-dimensional lattices predicts a convergence of the respective parameters (see Tables 3 ▸ and 6 ▸).

In a forthcoming paper (Simbrunner *et al.*, 2022 ▸) we will check our theoretically derived results by indexing only the *x* and *y* components of the reciprocal vectors obtained in our previous GIXD experiments. In a further step, we will compare these findings with the results of recent LEED experiments on the same molecules to compare the properties of monolayer and multilayer (Simbrunner *et al.*, 2022 ▸).

## Figures and Tables

**Table 1:** Explicit expressions for the parameters *a*′, *b*′ and γ′ of the reduced rhomboid, spanned by linear combinations of the vectors **a**, **b** and **c** with the coefficients λ_*i*_ and μ_*i*_, as functions of the parameters *a*, *b*, *c*, α, β and γ of the underlying parallelepiped, given for PTCDA/Ag(111), P2O/Ag(111), DCV4T-Et2/Ag(111) and *trans*-DBPen/Cu(111) The Miller indices (*uvw*) indicate the contact planes of the epitaxially oriented crystals. For experimental details see Simbrunner *et al.* (2020 ▸, 2021*a* ▸).

(*uvw*)	Coefficients λ, μ†	Cell parameters
PTCDA/Ag(111)		
103	λ_*a*_ = 0, λ_*b*_ = −1, λ_*c*_ = 0	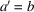
	μ_*a*_ = 3, μ_*b*_ = 0, μ_*c*_ = −1	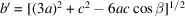
		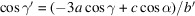
P2O/Ag(111)		
102	λ_*a*_ = 0, λ_*b*_ = −1, λ_*c*_ = 0	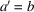
	μ_*a*_ = 2, μ_*b*_ = 0, μ_*c*_ = −1	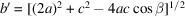
		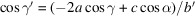
DCV4T-Et2/Ag(111)		
122	λ*_a_* = 0, λ*_b_* = −1, λ*_c_* = −1	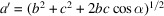
	μ*_a_* = 2, μ*_b_* = 1, μ*_c_* = 0	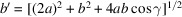
		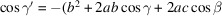 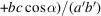
211	λ*_a_* = 0, λ*_b_* = −1, λ*_c_* = −1	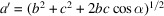
	μ*_a_* = 1, μ*_b_* = 1, μ*_c_* = −1	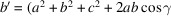 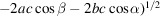
		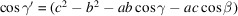 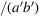
020	λ*_a_* = −1, λ*_b_* = 0, λ*_c_* = 0	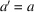
	μ*_a_* = 0, μ*_b_* = 0, μ*_c_* = 1	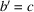
		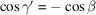
*trans*-DBPen/Cu(111)		
020	λ*_a_* = −1, λ*_b_* = 0, λ*_c_* = 0	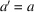
	μ*_a_* = 0, μ*_b_* = 0, μ*_c_* = 1	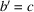
		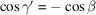

† *cf*. Equation (15).

**Table 2:** Parameters of the three-dimensional unit cells in PTCDA/Ag(111), P2O/Ag(111), DCV4T-Et2/Ag(111) and *trans*-DBPen/Cu(111), found experimentally by rotated GIXD experiments (Simbrunner *et al.*, 2020 ▸, 2021*a* ▸) The Miller indices (*uvw*) indicate the contact planes of the epitaxially oriented crystals.

(*uvw*)	*a* (Å)	*b* (Å)	*c* (Å)	α (°)	β (°)	γ (°)
PTCDA/Ag(111)						
(103) (103)	3.737 (7)	12.206 (102)	17.013 (90)	89.87 (12)	84.93 (28)	89.93 (6)
P2O/Ag(111)						
(102) (102)	5.059 (12)	8.097 (26)	8.916 (32)	91.64 (24)	92.95 (56)	94.17 (23)
DCV4T-Et2/Ag(111)						
(122) (122)	8.408 (17)	9.070 (14)	10.370 (12)	104.79 (10)	109.91 (6)	105.43 (8)
(211) (211)	8.083 (19)	8.401 (18)	9.860 (49)	97.74 (36)	93.57 (36)	92.49 (27)
(020) (020)	6.115 (9)	7.290 (9)	16.095 (13)	83.44 (20)	89.52 (17)	71.53 (13)
*trans*-DBPen/Cu(111)						
(020) (020)	6.751 (8)	7.566 (4)	18.529 (41)	89.88 (8)	86.71 (25)	89.84 (12)

**Table 3:** Parameters *a*′, *b*′, γ′ and ‘Area’ of the surface unit cells in the studied molecules, calculated from the parameters of the three-dimensional unit cells obtained from rotated GIXD experiments (Simbrunner *et al.*, 2020 ▸, 2021*a* ▸) The (calculated propagated) uncertainties are given in brackets. The composition of the unit-cell vectors **a**′ and **b**′ is indicated by the coefficients λ_*i*_ and μ_*i*_. The Miller indices (*uvw*) indicate the contact planes of the epitaxially oriented crystals.

(*uvw*)	**a**′, **b**′ [λ_*a*_ λ_*b*_ λ_*c*_], [μ_*a*_ μ_*b*_ μ_*c*_]	*a*′ (Å)	*b*′ (Å)	γ′ (°)	Area calculated† (Å^2^)
PTCDA/Ag(111)					
103	[010], [301]	12.206 (102)	19.530 (89)	89.93 (11)	238.4 (23)
P2O/Ag(111)					
102	[010], [201]	8.097 (26)	13.826 (70)	88.01 (23)	111.9 (7)
DCV4T-Et2/Ag(111)					
122	[01 1], [210]	11.907 (11)	16.849 (32)	78.00 (11)	196.2 (4)
211	[01 1], [111]	12.062 (56)	16.108 (62)	79.76 (32)	191.2 (12)
020	[100], [001]	6.115 (9)	16.095 (13)	90.48 (17)	98.4 (2)
*trans*-DBPen/Cu(111)					
020	[100], [001]	6.751 (8)	18.529 (41)	93.29 (25)	124.9 (3)

† *cf.* Equation (21).

**Table 4:** Area of the two-dimensional unit cells for PTCDA/Ag(111), P2O/Ag(111), DCV4T-Et2/Ag(111) and *trans*-DBPen/Cu(111), calculated from the specular scan in X-ray diffraction and the volume from GIXD, compared with the areas obtained from GIXD experiments (Simbrunner *et al.*, 2020 ▸, 2021*a* ▸)

Molecule/substrate	Miller indices (*uvw*)	*q*_spec_ (Å^−1^)	Vol. (Å^3^)	Area calculated† (Å^2^)
PTCDA/Ag(111)	±(103)	1.947 (2)	773.0 (28)	239.5 (9)
P2O/Ag(111)	±(102)	1.942 (2)	363.5 (4)	112.3 (2)
DCV4T-Et2/Ag(111)	±(122)	1.857 (2)	662.5 (14)	195.8 (5)
	±(211)	1.828 (2)	661.1 (36)	192.3 (11)
	±(020)	1.828 (2)	673.5 (13)	98.0 (2)‡
*trans*-DBPen/Cu(111)	±(020)	1.660 (2)	944.8 (13)	124.8 (2)‡

† *cf.* Equation (22).

‡ gcd = 2.

**Table 5:** Correlations between the diagonals in the three-dimensional lattice and the parameters of the two-dimensional unit cell for the ±(122) and ±(211) orientations in DCV4T-Et2/Ag(111) (Simbrunner *et al.*, 2021*a* ▸) In addition to the corresponding mathematical expressions, the calculated numbers from the three-dimensional unit cells are itemized. The respective propagated uncertainties are given in brackets.

Diagonal	3D lattice	2D lattice	Calculated (Å)
±(122)			
diag(2**a**,**b**) short	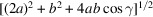	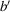	16.849 (32)
diag(2**a**,**c**) long	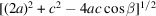	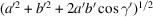	22.563 (38)
diag(**b**,**c**) short	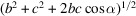	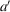	11.907 (11)
±(211)			
diag(**a**,2**b**) short	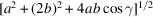	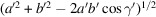	18.326 (84)
diag(**a**,2**c**) long	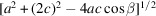	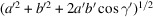	21.773 (77)
diag(**b**,**c**) short	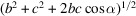	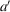	12.062 (56)

**Table 6:** Values of (ϕ_+_ − ϕ_−_) and Ω_λ,*uvw*_, calculated [using equation (58) and considering 60° symmetry] from the experimentally obtained parameters of our GIXD experiments on DCV4T-Et2/Ag(111), P2O/Ag(111), PTCDA/Ag(111) and *trans*-DBPen/Cu(111) (Simbrunner *et al.*, 2020 ▸, 2021*a* ▸) Also shown are the experimentally obtained values of φ_*uvw*_ − φ_−*u*−*v*−*w*_, where ±(*uvw*) are the corresponding contact planes.

Molecule/substrate	(*uvw*)	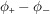	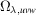	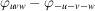
DCV4T-Et2/Ag(111)	(122)	+15.5 (5)°	282.0 (2)°	−8.5 (5)°
	(211)	+15.3 (4)°	239.9 (3)°	+15.4 (3)°
	(020)	+15.7 (5)°	0°	+15.7 (5)°
P2O/Ag(111)	(102)	−14.1 (10)°	0°	−14.1 (10)°
PTCDA/Ag(111)	(103)	±44.1 (12)°	0°	±44.1 (12)°
*trans*-DBPen/Cu(111)	(020)	+7.1 (4)°	0°	+7.1 (4)°

## Appendix A Determining the components of the two-dimensional unit-cell matrix

Assuming a contact plane with the Miller indices *u*, *v* and *w*, the matrix of the lattice vectors **A** can be written as (Simbrunner *et al.*, 2018 ▸)

where

and the angle φ represents a phase shift in the *xy* plane counter-clockwise.

Then the composed vector  with the relation  can be written as

where

and

where

For λ*_a_* = −*v*, λ*_b_* = *u* and λ*_c_* = 0, one obtains sin Ω_λ_ = 0 and cos Ω_λ_ = 1, resulting in Ω_λ_ = 0.

For λ*_a_* = −*w*, λ*_b_* = 0 and λ*_c_* = *u*, the following relations result:

where

For λ*_a_* = 0, λ*_b_* = −*w* and λ*_c_* = *v*, the following relations result:

where

For **b**′, the following expression is valid:

as [*cf*. equations (19), (42) and (53)]

Therefore, equation (22) can be explicitly written as

Equation (55) corresponds to equation (22) with the relation ϕ = φ + ψ + Ω_λ_. Note that for (*uvw*) → −(*uvw*) : (λ_*a*_, λ_*b*_, λ_*c*_) → (λ_*a*_, λ_*b*_, λ_*c*_) and (μ_*a*_, μ_*b*_, μ_*c*_) → −(μ_*a*_, μ_*b*_, μ_*c*_). For the angles Ω_λ_, Ω_μ_ and ψ of the contact plane (  ), the relations Ω_λ,−*u*−*v*−*w*_ = π − Ω_λ,*uvw*_, Ω_−μ,−*u*−*v*−*w*_ = 2π − Ω_μ,*uvw*_ and ψ_−*u*−*v*−*w*_ = π + ψ_*uvw*_ are valid, where Ω_λ,*uvw*_, Ω_μ,*uvw*_ and ψ_*uvw*_ are the respective angles of the contact plane (*uvw*). Consequently, Ω_−μ,−*u*−*v*−*w*_ − Ω_λ,−*u*−*v*−*w*_ = π − (Ω_μ,*uvw*_ − Ω_λ,*uvw*_). Hence, for (*uvw*) → −(*uvw*): γ′→ π − γ′ [see equation (54)]. Regarding equation (55), this is mathematically equivalent to γ′→ −γ′ and *h*′ → −*h*′, resulting in a mirror symmetry about an axis along the lattice vector **a**′.

Furthermore, the following relations can be deduced:

and

where  and  are the angles of the corresponding unit cells with mirror symmetry. Then,

In Table 6 ▸, the predicted values for  , obtained from our previous GIXD experiments (Simbrunner *et al.*, 2020 ▸, 2021*a* ▸), are shown. Note, that for the three polymorphs of DCV4T-Et2/Ag(111) these values are similar.

Using equations (47) and (48), the following equation can be deduced:

Using equation (15), the following expression can be derived:

where gcd(*u*, *v*, *w*) is the greatest common divisor of the Miller indices. For the area of the rhomboid, the following relations are valid:

## Appendix B Further mathematical expressions if *u*, *v* and *w* ≠ 0

From the equations (26)–(28), which are valid for *u*, *v* and *w* ≠ 0, further expressions for linear combinations of the vectors **a**, **b** and **c** can be derived. Considering the vector combinations (*v* **a** − *u* **b**), (*w* **a** − *u* **c**) and (*w* **b** − *v* **c**), their lengths and their corresponding angles can be determined, if the following expressions are known:

The right sides of equations (62)–(64) correspond to the left sides of equations (26)–(28) and hence can be determined from the parameters of the two-dimensional unit cell *a*′, *b*′ and γ′. For the equations (65)–(67) the following relations are useful:

and
